# 6-(4-Fluoro­pheneth­yl)-7-imino-3-phenyl-2,3,6,7-tetra­hydro-1,3-thia­zolo[4,5-*d*]pyrimidine-2-thione

**DOI:** 10.1107/S1600536809047576

**Published:** 2009-11-14

**Authors:** Ying Liang, Hong-Wu He, Zi-Wen Yang

**Affiliations:** a Hubei Biopesticide Engineering Research Center, Hubei Academy of Agricultural Science, Wuhan 430064, People’s Republic of China; b Key Laboratory of Pesticides & Chemical Biology of the Ministry of Education, Central China Normal University, Wuhan 430079, People’s Republic of China

## Abstract

In the title compound, C_19_H_15_FN_4_S_2_, the mean plane of the thia­zolopyrimidine makes a dihedral angle of 77.6 (1)° with the attached phenyl ring. The crystal packing is stabilized by inter­molecular C—H⋯N hydrogen bonds and weak C—H—π stacking inter­actions.

## Related literature

For the biological activity of thia­zolo[4,5-*d*]pyrimidine deriv­atives, see: Balkan *et al.* (2002 ▶); Bekhit *et al.* (2003 ▶); Danel *et al.* (1998 ▶); Fahmy *et al.* (2003 ▶). For the synthesis of thia­zolo [4,5-*d*]pyrimidines *via* tandem aza-Wittig and cyclization reactions of imino­phospho­rane and alkyl­amines, see: Liang *et al.* (2007 ▶). For C—H⋯π inter­actions, see: Janiak (2000 ▶). For bond-length data, see: Allen *et al.* (1987 ▶).
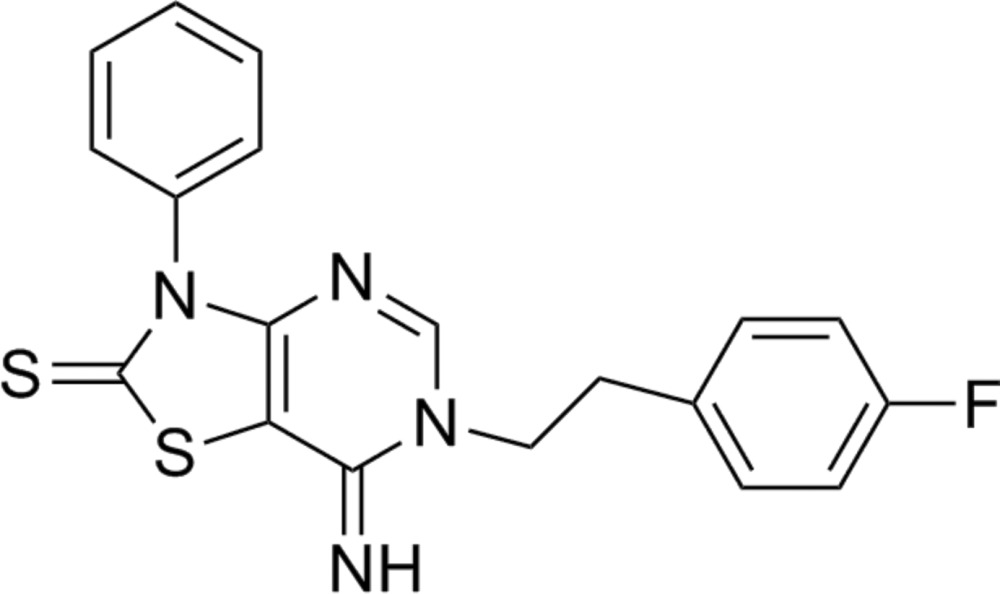


## Experimental

### 

#### Crystal data

C_19_H_15_FN_4_S_2_
*M* *_r_* = 382.47
Monoclinic, 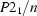

*a* = 8.6449 (13) Å
*b* = 12.3780 (19) Å
*c* = 16.546 (3) Å
β = 91.531 (3)°
*V* = 1769.9 (5) Å^3^
*Z* = 4
Mo *K*α radiation
μ = 0.32 mm^−1^
*T* = 298 K
0.30 × 0.20 × 0.20 mm

#### Data collection

Bruker SMART APEX CCD area-detector diffractometer
Absorption correction: multi-scan (*SADABS*; Sheldrick, 2001 ▶) *T*_min_ = 0.910, *T*_max_ = 0.939
13207 measured reflections
4047 independent reflections
3442 reflections with *I* > 2σ(*I*)
*R*_int_ = 0.023

#### Refinement

*R*[*F* ^2^ > 2σ(*F* ^2^)] = 0.045
*wR*(*F* ^2^) = 0.119
*S* = 1.05
4047 reflections
238 parameters
H atoms treated by a mixture of independent and constrained refinement
Δρ_max_ = 0.31 e Å^−3^
Δρ_min_ = −0.24 e Å^−3^

### 

Data collection: *SMART* (Bruker, 2000 ▶); cell refinement: *SAINT* (Bruker, 2000 ▶); data reduction: *SAINT*; program(s) used to solve structure: *SHELXS97* (Sheldrick, 2008 ▶); program(s) used to refine structure: *SHELXL97* (Sheldrick, 2008 ▶); molecular graphics: *SHELXTL* (Sheldrick, 2008 ▶); software used to prepare material for publication: *SHELXTL*.

## Supplementary Material

Crystal structure: contains datablocks global, I. DOI: 10.1107/S1600536809047576/jh2113sup1.cif

Structure factors: contains datablocks I. DOI: 10.1107/S1600536809047576/jh2113Isup2.hkl

Additional supplementary materials:  crystallographic information; 3D view; checkCIF report

## Figures and Tables

**Table 1:** Hydrogen-bond geometry (Å, °)

*D*—H⋯*A*	*D*—H	H⋯*A*	*D*⋯*A*	*D*—H⋯*A*
C15—H15⋯N3^i^	0.93	2.61	3.486 (3)	156
C19—H19⋯*Cg*3^ii^	0.93	2.74	3.637 (2)	161

Symmetry codes: (i) 
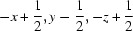
; (ii) 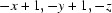. *Cg*3 is the centroid of the C1–C6 ring.

**Fig. 1.**
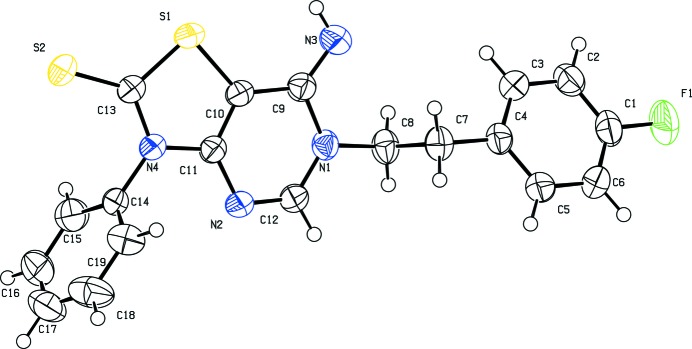
The structure of (I), showing 50% probability displacement ellipsoids and the atom-numbering scheme.

**Fig. 2.**
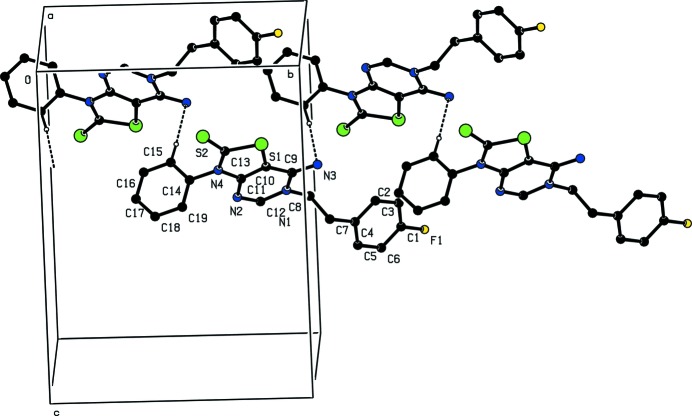
Crystal Packing diagram of (I). Hydrogen bonds are shown as dashed lines.

## supplementary crystallographic information

### Comment

Thiazolo[4,5-*d*]pyrimidine derivatives, which can be considered as thia-analogues of the natural purine bases such as adenine and guanine, have acquired a growing importance as anticancer agents (Fahmy *et al.*, 2003), antiviral agents used in the treatment of human cytomegalovirus (Bekhit *et al.*, 2003), antitumour agents (Balkan *et al.*,2002) and antibacterial agents (Danel *et al.*, 1998).

An important synthetic route of our previous reports for thiazolo [4,5-*d*]pyrimidines is the tandem aza-Wittig and cyclization reaction of iminophosphorane and alkylamines (Liang *et al.*, 2007). Recently, we have developed a new cyclization process to synthesize novel thiazolo[4,5-*d*]pyrimidine derivatives. In this paper, we report the structure of the title compound, (I)(Fig. 1).

In the molecule, all bond lengths and angles are normal (Allen *et al.*, 1987). The mean plane of the thiazolopyrimidine fragment makes dihedral angle of 77.58 (10)° with the attached phenyl ring fragment. In the crystal structure, intermolecular C—H···N hydrogen-bonding interactions stabilize the structure (Table 1). In addition, short intermolecular distances between the centroids of the C1···C6 ring, *Cg*3, and C19···H19A [C19—H19···*Cg*3^i^ = 2.740 (3) Å; symmetry code: (i) 1 - *x*, 1 - *y*, -*z*] indicate the existence of C—H-π stacking interactions (Janiak, 2000), which stabilize the crystal packing (Fig. 2) together with hydrogen-bonding interactions.

### Experimental

To a suspension of 5-cyano-4-ethoxymethyleneamino-3-phenyl-3*H*-thiazolin- 2-thione (0.87 g 5 mmol) in 15 mL dry acetonitrile was added all at once 0.5 g (3.6 mmol) 4-fluorophenylethylamine. After standing at room temperature for 1.5 h, then the solution concentrated under vacuum and the residue was recrystallized from dichloromethane to give the title compound (yield 72.8%). Colourless crystals of (I) suitable for X-ray structure analysis were grown from the mixture of dichloromethane and ethanol (*v*/*v*, 1:3).

### Refinement

All H-atoms bound to carbon were refined using a riding model with d(C—H) = 0.93 Å, *U*_iso_=1.2*U*_eq_ (C) for aromatic 0.98 Å, *U*_iso_ = 1.2*U*_eq_ (C) for CH and 0.96 Å, *U*_iso_ = 1.5*U*_eq_ (C) for CH_3_ atoms.

### Crystal data

**Table:**

C_19_H_15_FN_4_S_2_	*F*(000) = 792
*M**_r_* = 382.47	*D*_x_ = 1.435 Mg m^−^^3^
Monoclinic, *P*2_1_/*n*	Mo *K*α radiation, λ = 0.71073 Å
*a* = 8.6449 (13) Å	Cell parameters from 5242 reflections
*b* = 12.3780 (19) Å	θ = 2.5–27.9°
*c* = 16.546 (3) Å	µ = 0.32 mm^−^^1^
β = 91.531 (3)°	*T* = 298 K
*V* = 1769.9 (5) Å^3^	Block, colorless
*Z* = 4	0.30 × 0.20 × 0.20 mm

### Data collection

**Table:**

Bruker SMART APEX CCD area-detector diffractometer	4047 independent reflections
Radiation source: fine-focus sealed tube	3442 reflections with *I* > 2σ(*I*)
graphite	*R*_int_ = 0.023
φ and ω scans	θ_max_ = 27.5°, θ_min_ = 2.1°
Absorption correction: multi-scan (*SADABS*; Sheldrick, 2001)	*h* = −11→11
*T*_min_ = 0.910, *T*_max_ = 0.939	*k* = −15→16
13207 measured reflections	*l* = −20→21

### Refinement

**Table:**

Refinement on *F*^2^	Primary atom site location: structure-invariant direct methods
Least-squares matrix: full	Secondary atom site location: difference Fourier map
*R*[*F*^2^ > 2σ(*F*^2^)] = 0.045	Hydrogen site location: inferred from neighbouring sites
*wR*(*F*^2^) = 0.119	H atoms treated by a mixture of independent and constrained refinement
*S* = 1.05	*w* = 1/[σ^2^(*F*_o_^2^) + (0.063*P*)^2^ + 0.3948*P*] where *P* = (*F*_o_^2^ + 2*F*_c_^2^)/3
4047 reflections	(Δ/σ)_max_ = 0.001
238 parameters	Δρ_max_ = 0.31 e Å^−^^3^
0 restraints	Δρ_min_ = −0.24 e Å^−^^3^

### Special details

**Table:**

Geometry. All e.s.d.'s (except the e.s.d. in the dihedral angle between two l.s. planes) are estimated using the full covariance matrix. The cell e.s.d.'s are taken into account individually in the estimation of e.s.d.'s in distances, angles and torsion angles; correlations between e.s.d.'s in cell parameters are only used when they are defined by crystal symmetry. An approximate (isotropic) treatment of cell e.s.d.'s is used for estimating e.s.d.'s involving l.s. planes.
Refinement. Refinement of *F*^2^ against ALL reflections. The weighted *R*-factor *wR* and goodness of fit *S* are based on *F*^2^, conventional *R*-factors *R* are based on *F*, with *F* set to zero for negative *F*^2^. The threshold expression of *F*^2^ > σ(*F*^2^) is used only for calculating *R*-factors(gt) *etc*. and is not relevant to the choice of reflections for refinement. *R*-factors based on *F*^2^ are statistically about twice as large as those based on *F*, and *R*- factors based on ALL data will be even larger.

### Fractional atomic coordinates and isotropic or equivalent isotropic displacement parameters (Å^2^)

**Table:**

	*x*	*y*	*z*	*U*_iso_*/*U*_eq_	
C1	0.4958 (2)	1.34258 (14)	0.58804 (13)	0.0535 (5)	
C2	0.4537 (2)	1.33892 (14)	0.50835 (13)	0.0542 (5)	
H2	0.4863	1.3917	0.4726	0.065*	
C3	0.3607 (2)	1.25414 (15)	0.48189 (11)	0.0502 (4)	
H3	0.3306	1.2503	0.4276	0.060*	
C4	0.31153 (19)	1.17484 (13)	0.53462 (11)	0.0429 (4)	
C5	0.3617 (2)	1.18055 (15)	0.61453 (11)	0.0504 (4)	
H5	0.3335	1.1265	0.6503	0.060*	
C6	0.4530 (2)	1.26536 (16)	0.64208 (12)	0.0572 (5)	
H6	0.4846	1.2697	0.6961	0.069*	
C7	0.2083 (2)	1.08369 (14)	0.50454 (11)	0.0482 (4)	
H7A	0.1203	1.1133	0.4745	0.058*	
H7B	0.1696	1.0439	0.5503	0.058*	
C8	0.29661 (19)	1.00780 (14)	0.45073 (12)	0.0467 (4)	
H8A	0.3450	1.0496	0.4087	0.056*	
H8B	0.3782	0.9732	0.4827	0.056*	
C9	0.10871 (19)	0.95138 (14)	0.34270 (10)	0.0422 (4)	
C10	0.03504 (19)	0.85689 (13)	0.30865 (10)	0.0411 (4)	
C11	0.04789 (18)	0.75959 (13)	0.34560 (9)	0.0379 (3)	
C12	0.1989 (2)	0.82329 (14)	0.44484 (11)	0.0458 (4)	
H12	0.2567	0.8135	0.4925	0.055*	
C13	−0.0990 (2)	0.70793 (14)	0.23294 (10)	0.0445 (4)	
C14	−0.02508 (18)	0.56612 (13)	0.33171 (10)	0.0402 (4)	
C15	0.0719 (3)	0.49373 (17)	0.29640 (13)	0.0621 (5)	
H15	0.1341	0.5146	0.2541	0.075*	
C16	0.0753 (3)	0.38912 (19)	0.32496 (16)	0.0780 (7)	
H16	0.1398	0.3386	0.3014	0.094*	
C17	−0.0147 (3)	0.35897 (17)	0.38718 (18)	0.0776 (8)	
H17	−0.0116	0.2881	0.4058	0.093*	
C18	−0.1101 (3)	0.4326 (2)	0.42259 (16)	0.0739 (7)	
H18	−0.1711	0.4117	0.4653	0.089*	
C19	−0.1156 (2)	0.53768 (16)	0.39494 (12)	0.0554 (5)	
H19	−0.1796	0.5882	0.4188	0.066*	
N1	0.19862 (15)	0.92398 (11)	0.41267 (8)	0.0411 (3)	
N2	0.12708 (17)	0.73932 (11)	0.41615 (9)	0.0459 (3)	
N3	0.1072 (2)	1.04959 (13)	0.31970 (11)	0.0605 (4)	
H3A	0.052 (3)	1.0564 (19)	0.2764 (15)	0.073*	
N4	−0.02702 (16)	0.67676 (11)	0.30352 (8)	0.0397 (3)	
F1	0.58230 (17)	1.42773 (10)	0.61527 (9)	0.0823 (4)	
S1	−0.07386 (6)	0.84673 (4)	0.21932 (3)	0.05356 (16)	
S2	−0.19713 (7)	0.63041 (4)	0.16868 (3)	0.06299 (18)	

### Atomic displacement parameters (Å^2^)

**Table:**

	*U*^11^	*U*^22^	*U*^33^	*U*^12^	*U*^13^	*U*^23^
C1	0.0557 (10)	0.0390 (9)	0.0653 (12)	−0.0015 (7)	−0.0051 (9)	−0.0124 (8)
C2	0.0649 (11)	0.0400 (9)	0.0579 (12)	−0.0030 (8)	0.0053 (9)	0.0022 (8)
C3	0.0616 (11)	0.0468 (10)	0.0418 (9)	0.0013 (8)	−0.0036 (8)	−0.0013 (7)
C4	0.0455 (9)	0.0374 (8)	0.0457 (9)	0.0022 (7)	0.0017 (7)	−0.0069 (7)
C5	0.0657 (11)	0.0424 (9)	0.0431 (10)	0.0013 (8)	0.0027 (8)	−0.0003 (7)
C6	0.0738 (13)	0.0516 (11)	0.0456 (10)	0.0017 (9)	−0.0113 (9)	−0.0088 (8)
C7	0.0463 (9)	0.0461 (9)	0.0523 (10)	−0.0049 (7)	0.0040 (8)	−0.0077 (8)
C8	0.0384 (8)	0.0437 (9)	0.0576 (11)	−0.0028 (7)	−0.0038 (7)	−0.0108 (8)
C9	0.0456 (9)	0.0420 (9)	0.0391 (8)	−0.0044 (7)	0.0005 (7)	0.0010 (7)
C10	0.0478 (9)	0.0415 (8)	0.0337 (8)	−0.0036 (7)	−0.0048 (7)	0.0028 (6)
C11	0.0403 (8)	0.0388 (8)	0.0344 (8)	−0.0022 (6)	−0.0019 (6)	−0.0006 (6)
C12	0.0493 (9)	0.0426 (9)	0.0448 (10)	0.0028 (7)	−0.0131 (7)	−0.0014 (7)
C13	0.0541 (10)	0.0429 (9)	0.0361 (8)	−0.0027 (7)	−0.0056 (7)	0.0005 (7)
C14	0.0455 (8)	0.0356 (8)	0.0390 (8)	−0.0024 (6)	−0.0096 (7)	−0.0015 (6)
C15	0.0763 (14)	0.0584 (12)	0.0516 (11)	0.0159 (10)	0.0021 (10)	−0.0024 (9)
C16	0.1057 (19)	0.0501 (12)	0.0772 (16)	0.0275 (12)	−0.0187 (14)	−0.0099 (11)
C17	0.0875 (16)	0.0422 (11)	0.101 (2)	−0.0070 (11)	−0.0404 (15)	0.0170 (11)
C18	0.0622 (12)	0.0699 (15)	0.0891 (17)	−0.0130 (11)	−0.0077 (11)	0.0346 (13)
C19	0.0493 (10)	0.0549 (11)	0.0621 (12)	0.0020 (8)	0.0031 (8)	0.0128 (9)
N1	0.0389 (7)	0.0388 (7)	0.0453 (8)	−0.0018 (5)	−0.0053 (6)	−0.0052 (6)
N2	0.0560 (8)	0.0401 (7)	0.0409 (8)	−0.0006 (6)	−0.0147 (6)	0.0024 (6)
N3	0.0840 (12)	0.0420 (8)	0.0547 (10)	−0.0113 (8)	−0.0114 (9)	0.0082 (7)
N4	0.0479 (7)	0.0373 (7)	0.0335 (7)	−0.0034 (5)	−0.0060 (5)	0.0010 (5)
F1	0.0964 (10)	0.0541 (7)	0.0954 (10)	−0.0209 (7)	−0.0147 (8)	−0.0206 (7)
S1	0.0767 (3)	0.0453 (3)	0.0377 (3)	−0.0082 (2)	−0.0170 (2)	0.00820 (18)
S2	0.0860 (4)	0.0531 (3)	0.0483 (3)	−0.0078 (2)	−0.0276 (3)	−0.0050 (2)

### Geometric parameters (Å, °)

**Table:**

C1—C2	1.359 (3)	C10—S1	1.7356 (16)
C1—F1	1.362 (2)	C11—N2	1.361 (2)
C1—C6	1.367 (3)	C11—N4	1.390 (2)
C2—C3	1.386 (3)	C12—N2	1.294 (2)
C2—H2	0.9300	C12—N1	1.355 (2)
C3—C4	1.387 (3)	C12—H12	0.9300
C3—H3	0.9300	C13—N4	1.364 (2)
C4—C5	1.382 (2)	C13—S2	1.6501 (17)
C4—C7	1.515 (2)	C13—S1	1.7472 (18)
C5—C6	1.384 (3)	C14—C15	1.369 (3)
C5—H5	0.9300	C14—C19	1.369 (3)
C6—H6	0.9300	C14—N4	1.447 (2)
C7—C8	1.515 (2)	C15—C16	1.378 (3)
C7—H7A	0.9700	C15—H15	0.9300
C7—H7B	0.9700	C16—C17	1.359 (4)
C8—N1	1.470 (2)	C16—H16	0.9300
C8—H8A	0.9700	C17—C18	1.371 (4)
C8—H8B	0.9700	C17—H17	0.9300
C9—N3	1.274 (2)	C18—C19	1.379 (3)
C9—N1	1.418 (2)	C18—H18	0.9300
C9—C10	1.439 (2)	C19—H19	0.9300
C10—C11	1.354 (2)	N3—H3A	0.86 (2)
			
C2—C1—F1	118.50 (18)	C10—C11—N2	125.78 (15)
C2—C1—C6	122.77 (17)	C10—C11—N4	113.49 (14)
F1—C1—C6	118.72 (18)	N2—C11—N4	120.72 (14)
C1—C2—C3	118.01 (18)	N2—C12—N1	126.76 (15)
C1—C2—H2	121.0	N2—C12—H12	116.6
C3—C2—H2	121.0	N1—C12—H12	116.6
C2—C3—C4	121.47 (17)	N4—C13—S2	127.02 (13)
C2—C3—H3	119.3	N4—C13—S1	109.46 (12)
C4—C3—H3	119.3	S2—C13—S1	123.52 (10)
C5—C4—C3	118.15 (16)	C15—C14—C19	121.81 (18)
C5—C4—C7	121.30 (16)	C15—C14—N4	118.95 (17)
C3—C4—C7	120.54 (16)	C19—C14—N4	119.19 (15)
C4—C5—C6	121.04 (18)	C14—C15—C16	118.4 (2)
C4—C5—H5	119.5	C14—C15—H15	120.8
C6—C5—H5	119.5	C16—C15—H15	120.8
C1—C6—C5	118.50 (18)	C17—C16—C15	120.7 (2)
C1—C6—H6	120.7	C17—C16—H16	119.7
C5—C6—H6	120.7	C15—C16—H16	119.7
C4—C7—C8	110.67 (14)	C16—C17—C18	120.3 (2)
C4—C7—H7A	109.5	C16—C17—H17	119.9
C8—C7—H7A	109.5	C18—C17—H17	119.9
C4—C7—H7B	109.5	C17—C18—C19	120.1 (2)
C8—C7—H7B	109.5	C17—C18—H18	120.0
H7A—C7—H7B	108.1	C19—C18—H18	120.0
N1—C8—C7	113.30 (14)	C14—C19—C18	118.7 (2)
N1—C8—H8A	108.9	C14—C19—H19	120.6
C7—C8—H8A	108.9	C18—C19—H19	120.6
N1—C8—H8B	108.9	C12—N1—C9	122.40 (13)
C7—C8—H8B	108.9	C12—N1—C8	119.05 (14)
H8A—C8—H8B	107.7	C9—N1—C8	118.54 (14)
N3—C9—N1	118.24 (15)	C12—N2—C11	113.08 (14)
N3—C9—C10	131.12 (16)	C9—N3—H3A	110.2 (16)
N1—C9—C10	110.63 (14)	C13—N4—C11	114.58 (13)
C11—C10—C9	121.06 (15)	C13—N4—C14	123.01 (13)
C11—C10—S1	110.87 (12)	C11—N4—C14	122.39 (12)
C9—C10—S1	128.06 (12)	C10—S1—C13	91.60 (8)
			
F1—C1—C2—C3	−177.55 (17)	C17—C18—C19—C14	0.3 (3)
C6—C1—C2—C3	1.4 (3)	N2—C12—N1—C9	3.5 (3)
C1—C2—C3—C4	−0.1 (3)	N2—C12—N1—C8	−175.59 (17)
C2—C3—C4—C5	−1.9 (3)	N3—C9—N1—C12	175.35 (18)
C2—C3—C4—C7	179.23 (16)	C10—C9—N1—C12	−5.8 (2)
C3—C4—C5—C6	2.7 (3)	N3—C9—N1—C8	−5.6 (2)
C7—C4—C5—C6	−178.48 (17)	C10—C9—N1—C8	173.26 (14)
C2—C1—C6—C5	−0.7 (3)	C7—C8—N1—C12	−100.26 (19)
F1—C1—C6—C5	178.28 (18)	C7—C8—N1—C9	80.6 (2)
C4—C5—C6—C1	−1.4 (3)	N1—C12—N2—C11	1.3 (3)
C5—C4—C7—C8	−109.01 (19)	C10—C11—N2—C12	−3.2 (3)
C3—C4—C7—C8	69.8 (2)	N4—C11—N2—C12	175.53 (15)
C4—C7—C8—N1	−173.93 (15)	S2—C13—N4—C11	179.73 (13)
N3—C9—C10—C11	−177.3 (2)	S1—C13—N4—C11	−0.73 (19)
N1—C9—C10—C11	4.0 (2)	S2—C13—N4—C14	1.2 (3)
N3—C9—C10—S1	4.0 (3)	S1—C13—N4—C14	−179.28 (13)
N1—C9—C10—S1	−174.59 (13)	C10—C11—N4—C13	0.2 (2)
C9—C10—C11—N2	0.3 (3)	N2—C11—N4—C13	−178.63 (15)
S1—C10—C11—N2	179.18 (14)	C10—C11—N4—C14	178.81 (15)
C9—C10—C11—N4	−178.47 (15)	N2—C11—N4—C14	−0.1 (2)
S1—C10—C11—N4	0.37 (19)	C15—C14—N4—C13	76.3 (2)
C19—C14—C15—C16	1.2 (3)	C19—C14—N4—C13	−106.2 (2)
N4—C14—C15—C16	178.58 (18)	C15—C14—N4—C11	−102.1 (2)
C14—C15—C16—C17	−0.5 (4)	C19—C14—N4—C11	75.4 (2)
C15—C16—C17—C18	−0.2 (4)	C11—C10—S1—C13	−0.66 (14)
C16—C17—C18—C19	0.3 (4)	C9—C10—S1—C13	178.08 (17)
C15—C14—C19—C18	−1.1 (3)	N4—C13—S1—C10	0.78 (14)
N4—C14—C19—C18	−178.46 (17)	S2—C13—S1—C10	−179.66 (13)

### Hydrogen-bond geometry (Å, °)

**Table:**

*D*—H···*A*	*D*—H	H···*A*	*D*···*A*	*D*—H···*A*
C15—H15···N3^i^	0.93	2.61	3.486 (3)	156
C19—H19···Cg3^ii^	0.93	2.74	3.637 (2)	161

Symmetry codes: (i) −*x*+1/2, *y*−1/2, −*z*+1/2; (ii) −*x*+1, −*y*+1, −*z*.
